# Organizational Virtuousness and Workplace Thriving: The Moderating Role of Workplace Ostracism Among Nurses

**DOI:** 10.3390/healthcare14213677

**Published:** 2026-10-30

**Authors:** Shimaa M. Salem, Hayam Ahmed Elshrief, Amal R. Gab Allah, Safaa Gaber Aly Salem, Marwa A. Shahin, Radwa Mohamed Said Haggag

**Affiliations:** 1 Department of Nursing Administration, Faculty of Nursing, Menoufia University, Shibin Al Kawm 32511, Menoufia, Egypt; shimaa_salem12@nursing.menofia.edu.eg (S.M.S.); hiam.elsherief@nursing.menofia.edu.eg (H.A.E.); radwa.haggag@nursing.menofia.edu.eg (R.M.S.H.); 2 Department of Community Health Nursing, Faculty of Nursing, Al-Baha University, Al-Baha 61008, Saudi Arabia; 3 Department of Maternity, Obst & Gyn Nursing, College of Nursing, Princess Nourah Bint Abdulrahman University, P.O. Box 84428, Riyadh 11671, Saudi Arabia; 4 Department of Maternity and Gynecological Nursing, Nursing Program, Batterjee Medical College, Jeddah 21442, Saudi Arabia; marwa.shahin@bmc.edu.sa

**Keywords:** nurses, organizational virtuousness, workplace thriving, workplace ostracism

## Abstract

Background: Organizational virtuousness fosters a self-reinforcing spiral of workplace thriving, which encompasses the joint experience of vitality and learning at work. Workplace ostracism refers to the exclusion or ignoring of individuals or groups within the workplace and may negatively affect employees’ workplace experiences. Aim: We aimed to examine the relationship between organizational virtuousness and workplace thriving and to explore the moderating role of workplace ostracism among nurses. Methods: A descriptive correlational research design was employed. A simple random sample of 464 nurses working in inpatient departments and critical care units at Shibin El-Kowm Teaching Hospital and El Helal Insurance Hospital was recruited. Data were collected using three instruments: the Organizational Virtuousness Scale, the Thriving from Work Questionnaire, and the Workplace Ostracism Scale. Results: Nearly half of the nurses reported a high level of organizational virtuousness, while more than half reported a moderate level of workplace thriving. In contrast, nearly two-thirds reported a low level of workplace ostracism. Organizational virtuousness was significantly and positively correlated with workplace thriving and significantly and negatively correlated with workplace ostracism. Furthermore, workplace ostracism significantly moderated the relationship between organizational virtuousness and workplace thriving. Conclusions: Organizational virtuousness was positively associated with workplace thriving among nurses, whereas workplace ostracism was negatively associated with organizational virtuousness. Furthermore, the significant interaction between organizational virtuousness and workplace ostracism indicated that the strength of the association between organizational virtuousness and workplace thriving varied across levels of workplace ostracism. Implications for Nursing Practice: Nurse managers should foster supportive work environments by providing tangible and emotional support to strengthen organizational virtuousness and promote workplace thriving. Nursing leaders should also implement proactive strategies to identify and address workplace ostracism and create an inclusive environment in which nurses feel valued, respected, and supported.

## 1. Introduction

Nurses work in complex and demanding healthcare environments in which organizational conditions and interpersonal relationships can substantially shape their experiences at work. Beyond the provision of safe and effective patient care, nurses increasingly need supportive work environments that enable them to remain energized, learn from their experiences, develop professionally, and maintain positive functioning in the workplace. Organizational practices and the quality of interactions among colleagues may therefore play an important role in creating work environments in which nurses can thrive. Conversely, negative interpersonal experiences, such as being ignored, excluded, or socially isolated by colleagues, may undermine nurses’ positive experiences and hinder their ability to function and develop effectively at work [1].

In the past decades, several cases of corporate misconduct have led managers to realize the importance of “virtues” in organizational contexts [2,3]. Organizational virtuousness leads to reduced workplace ostracism, enhanced organizational performance, increased nurses’ job satisfaction, greater workplace thriving, civic engagement, and affective commitment [4]. Positive social interactions among coworkers enhance nurse engagement and well-being, fostering a collective drive for group benefits within the organization [5].

Organizational virtue involves behaviors and procedures that promote virtuousness within an organization [6]. Individuals recognize this virtue when they relate organizational practices to positive social impacts and moral values [7]. The goal is to attain the highest human objectives, reflecting moral principles that foster social harmony [2,8].

Organizational virtuousness has been described as an organizational context characterized by optimism, compassion, forgiveness, trust and integrity [5]. Existing studies have emphasized that organizational virtuousness can be fostered by promoting shared ethical values, common purpose and cooperation between individuals [9]. Furthermore, encouraging cooperation among people, a shared goal, and ethical principles can all help to cultivate organizational virtue [9]. Virtues that stand for strength or excellence include transcendence, temperance, justice, humanity, courage, and wisdom. These characteristics and behaviors reflect intellectual, moral, and social excellence and are intended to advance human goodness and attain an ideal state of functioning [10].

Organizational virtuousness and workplace thriving have proved to produce desired organizational outcomes like good performance [11]. One significant strategy to increase workplace thriving in a healthcare organization is to increase nurses’ satisfaction. Nurse loyalty, increased workplace thriving, lower workplace ostracism, and greater dedication are more likely to be inspired by leaders who actively foster a virtuous culture and model virtuous behavior. Virtues are the habits, desires, and actions that generate individual and social well-being [12].

Organizational virtuousness encompasses a variety of aspects, including humanity, forgiveness, integrity, and trust [12]. Additionally, it includes the following aspects: organizational solidarity, organizational trust, organizational optimism, organizational compassion, and organizational mercy [5]. Additionally, optimism, trust, forgiveness, integrity, and compassion are the five elements that comprise organizational virtue [13].

Perception of organizational virtuousness promotes a self-reinforcing spiral of thriving, which is defined as a psychological experience combining vitality and learning [14]. Positive workplace environments cultivate these elements, where vitality represents energy at work and learning encompasses knowledge and skill development. This duality allows individuals to adapt and reshape their work environments, facilitating personal and professional growth [15].

Thriving at work has been significantly explored in the past decade [16,17]; workplace thriving is a multifaceted concept that includes both growth and emotional states and is related to well-being [14]. It entails handling stressful situations well, improving nurses’ physical and mental health, and fostering their personal development while having a positive effect on absenteeism and turnover [15]. In order to improve nursing quality and promote career development, thriving is essential [18].

High levels of thriving at work allow nurses to better adapt to their surroundings, which improves their physical and mental well-being. Such flourishing promotes the stability of healthcare systems and is essential for resilience against work pressures. Additionally, it results in improved work procedures, better care, and a stronger emphasis on patient safety [19].

Organizational virtue includes good moral and ethical behavior, whereas workplace ostracism is the exclusion or disdain of specific nurses or groups. Being virtuous improves relationships and teamwork among participants, creating a positive culture with qualities like trust and mercy. Because it goes against the organization’s values, this setting might lessen instances of exclusion [20].

Integrity, justice, and compassionate leaders foster organizational virtue, which benefits nurses with high psychological capital and increases thriving. Self-assurance in one’s abilities enhances learning, adaptability, and workplace vitality [21]. A moderating variable determines the direction and strength of a relationship, whereas a mediating variable explains the relationship between two variables. While multiple regression analysis looks at moderation, mediation analysis uses linear regression or ANOVAs to test whether a variable acts as a mediator [22].

The feeling of being unwanted in social settings at work is known as “workplace ostracism”. This phenomenon results from people feeling excluded or ignored by their colleagues, which decreases their productivity and emotional health. Ostracism can seriously impair job satisfaction and mental health in the nursing profession, where collaboration is crucial for the best possible patient care [23].

Workplace ostracism is acknowledged as a rather widespread phenomenon [24,25] that significantly and negatively influences both nurses and organizations [26]. Thriving at work is a contextual phenomenon in which workplace ostracism can portray a substantial negative role as a potential barrier [27], confirming its negative correlation with thriving at work [28]. Therefore, by integrating the conservation of resources theory (COR) [29] and the socially embedded model of thriving at work [30] it is suggested that nurses who experience ostracism within the workplace have depleted personal resources, thus hindering their ability to thrive at work and consequently engage in beneficial nursing and organizational outcomes [31].

Ostracism is different from other deviant behaviors, such as bullying, sexual harassment, and abusive supervision, because it includes undesirable behavior rather than direct intent to cause harm to others [32]. Social exclusion in an organizational setting is known as “workplace ostracism,” and it is typified by actions like coworkers refusing to extend an invitation, ignoring small talk, and avoiding eye contact [33,34]. This phenomenon is often studied in conjunction with other abnormal workplace behaviors because it frequently results in negative emotions like grief, rage, and hurt feelings [35].

Workplace ostracism encompasses subtle behaviors such as excluding people from social events and ignoring them during meetings, which can make them feel irrelevant and invisible [36]. The target’s well-being, job satisfaction, and productivity at work may all suffer as a result of this exclusion, creating a poisonous and upsetting atmosphere [37,38].

Ostracism can make nurses feel underappreciated by their employer, which lowers morale and motivation [39]. This disengagement can lead to psychological problems like anxiety and hopelessness, along with a decline in productivity, creativity, and general well-being at work [40,41,42].

Ostracized nurses are less engaged in their work, which undermines commitment to virtuous organizational values. This ostracism impedes teamwork, collaboration, and overall collective performance, negatively impacting workplace thriving. Furthermore, it has a negative impact on nurses’ well-being and participation in virtuous organizational practices, resulting in reduced operational efficacy and workplace thriving [12,38,43].

Although previous studies have examined organizational virtuousness and workplace ostracism in relation to various nurses’ outcomes, important gaps remain in understanding how these constructs are interconnected with workplace thriving, particularly within nursing settings. Recent research has examined the relationship between organizational virtuousness and workplace ostracism in relation to job performance and turnover intentions, including studies that have considered the moderating role of workplace ostracism or organizational virtuousness [12,20]. However, the emerging nursing literature indicates a need for greater theoretical integration of workplace ostracism with organizational and positive work-related factors and for examining these relationships within the distinctive interpersonal context of nursing work [44].

Accordingly, the present study extends previous research by integrating organizational virtuousness, workplace ostracism, and workplace thriving within a single analytical framework and by examining whether workplace ostracism moderates the association between organizational virtuousness and workplace thriving among nurses. Theoretically, the study draws on the Job Demands–Resources model to conceptualize organizational virtuousness as a potentially supportive organizational resource and workplace ostracism as a relevant social or interpersonal workplace demand. This integrated perspective advances current knowledge by considering how positive organizational conditions and negative interpersonal workplace experiences are simultaneously associated with nurses’ workplace thriving.

### 1.1. Significance of the Study

Organizations’ virtues are underexplored in the current literature, hindering their adoption. A positive perception of organizational virtuousness fosters a self-reinforcing cycle of workplace thriving, integrating vitality and learning. This virtuousness enhances overall thriving, motivates nurses to support the organization, promotes positive human outcomes, and mitigates negative attitudes among nurses [45].

This study fills a number of significant gaps in the body of the previous literature. First, there is not a lot of previous study on organizational virtuousness and how it relates to workplace thriving. Second, workplace ostracism’s insertion as a moderating role deepens our knowledge of the relationship between virtuousness and workplace thriving. Finally, the identification of workplace ostracism as a moderating factor is a noteworthy contribution. Recognizing the harmful influence of ostracism on the moderation role underscores the need for organizations to address and mitigate such negative workplace behaviors. Based on the above-mentioned information, this study aims to determine the relation between organizational virtuousness and workplace thriving and explore the moderating role of workplace ostracism as perceived by nurses.

### 1.2. Hypothesis Development

Organizational virtuousness encompasses five dimensions: optimism, trust, forgiveness, integrity, and compassion. Optimism reflects members’ belief in their ability to succeed in doing well, even under challenges. Trust indicates a respectful and considerate workplace environment. Forgiveness allows for mistakes to be seen as learning opportunities. Compassion involves caring and concern among coworkers [13]. Integrity, which promotes positive workplace behaviors and greatly improves performance, includes honor, dependability, and honesty within an organization [21,24].

Vitality, or the energy felt at work, and learning, or gaining knowledge and skills for personal development, are the two primary components of thriving, a positive psychological experience. The cognitive side of thriving is represented by learning, and the affective side is represented by vitality. This idea allows people to adapt and change their work environments [30].

According to the socially embedded model of thriving, nurses’ experiences of thriving are greatly influenced by contextual elements at the unit level, such as a supportive and respectful environment [30]. Nurses’ self-development is enhanced by practices and policies that promote organizational virtuousness, which acts as a contextual factor that supports both individual and organizational flourishing [21].

Organizational virtuousness has a contagion effect that encourages individuals to thrive and become more virtuous [21]. Trust, optimism, forgiveness, and integrity within organizations help break communication barriers and foster teaching. Compassionate practices toward employees can diminish negative behaviors and enhance their energy to meet goals [3,13]. Kaur and Ren (2026) [15] found that organizational virtuousness positively related to workplace thriving. Hence, H1 was proposed:

**H1.**  *Organizational virtuousness is positively related to workplace thriving.*

Excluding or ignoring someone at work is known as workplace ostracism, and it is particularly damaging for a number of reasons [24]. First of all, since people spend a large portion of their day interacting with coworkers, exclusion has a greater negative effect on their mental health [43]. Such exclusion can cause feelings of loneliness, anxiety, depression, and low self-esteem, all of which can have an impact on mental health and possibly cause absenteeism or decreased productivity [46].

Secondly, ostracism in the workplace negatively affects professional life by hindering participation in crucial meetings and projects, and limiting skill development and career growth. It leads to feelings of undervalue, resulting in decreased job satisfaction. Thirdly, cooperation and teamwork are vital in the workplace, as ostracism can erode trust and team cohesion, leading to difficulties in collaboration, a hostile environment, and decreased group performance [47,48].

Fourthly, ostracism can severely affect workplace culture, undermining employee confidence in the management and the organization. This exclusion may lead to a toxic environment, increased turnover, and decreased morale [49,50]; building on the dimensions of OV may reduce the ill effect of ostracism in the workplace. Gogia et al., (2024) [12] showed that organizational virtuousness negatively related to workplace ostracism. It is therefore hypothesized that:

**H2.**  *Organizational virtuousness is negatively related to workplace ostracism.*

Individual thriving, reflecting a perceived meaningful existence and growth at work, can be negatively impacted by workplace exclusion. Thriving encompasses cognitive (learning) and affective (vitality) aspects of personal development, where learning involves self-cognition and the progression in the workplace through knowledge and skills acquisition and application [30,51].

Vitality, which is essential for succeeding in the workplace, includes a person’s sense of energy and vitality. Despite their enthusiasm, nurses will struggle if they do not have opportunities to advance and develop their skills. On the other hand, constant learning results in stagnation if there is insufficient energy. Learning and vitality work together to foster thriving, which benefits both nurses and quality of work [16,51,52]. Wang, Y. (2023) [50] found that workplace ostracism is negatively related to workplace thriving. Therefore, the following hypothesis was suggested:

**H3.**  *Workplace ostracism is negatively related to workplace thriving.*

A framework for examining resource exchanges and social interactions, including both material and intangible exchanges between people or parties, is provided by social exchange theory (SET), which was created by Richard and Emerson in 1976 [53]. In this study, we explore how organizations engage in mutually beneficial activities through a model rooted in social exchange theory. It highlights the interplay between positive behaviors, fostering a thriving and ethical environment, and negative behaviors like workplace ostracism, which undermine job performance. Positive interactions nurture duty and trust, whereas exclusion diminishes these feelings, ultimately reducing productive organizational behavior [54].

Drawing on the social exchange theory, by fostering ethical and encouraging work environments, organizational virtue improves nurses’ workplace well-being. However, by restricting social support and resources, workplace exclusion can undermine these advantages and serve as a boundary condition that impacts the connection between thriving and virtue. Multiple research studies found thatworkplace ostracism moderates the relationship between organizational virtuousness and workplace thriving [12,38,43]. H4 was formulated as the following:

**H4.**  *Workplace ostracism moderates the relationship between organizational virtuousness and workplace thriving.*

**Figure 1:**
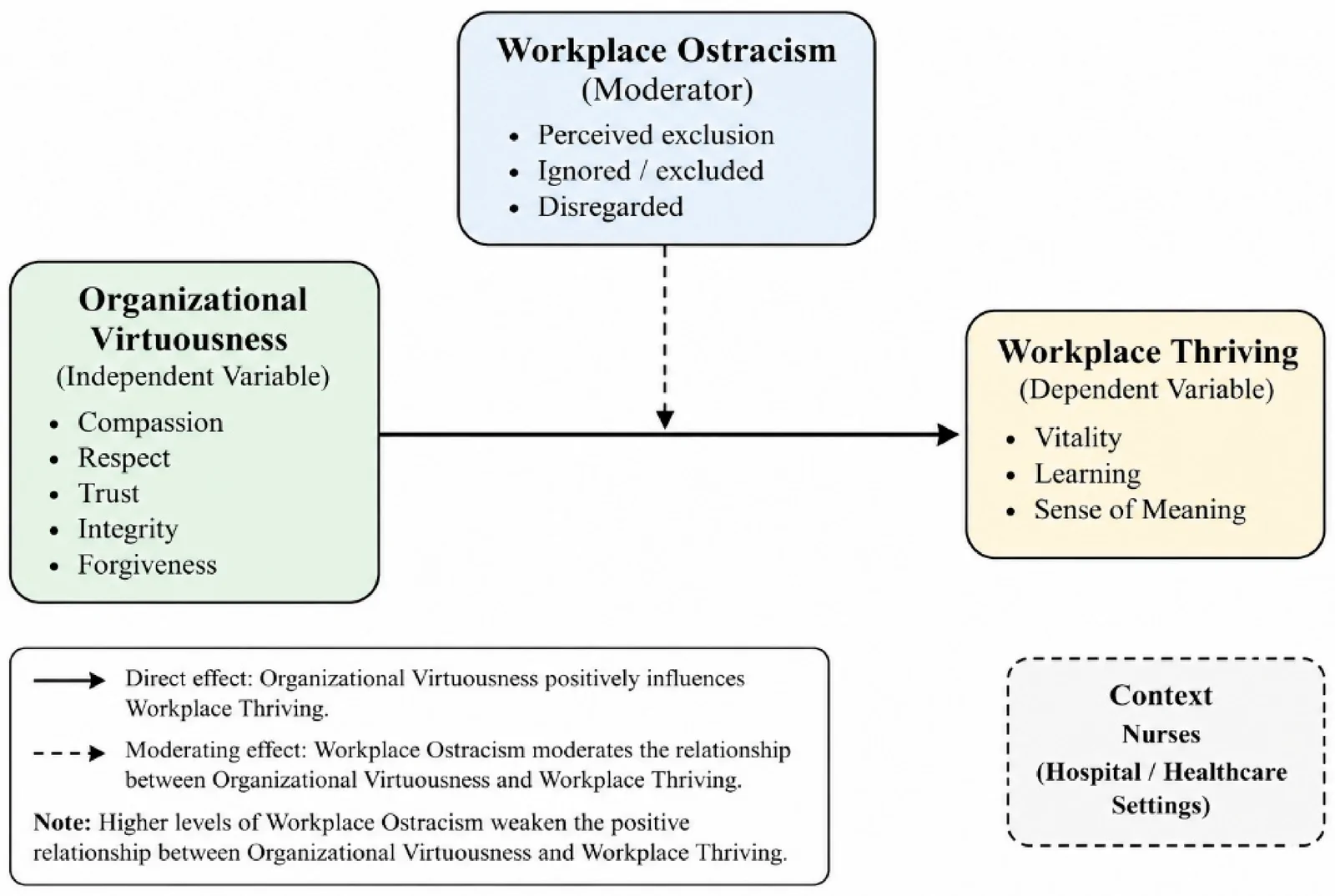
Hypothesized model.

Although previous research has established significant relationships between positive organizational conditions, workplace ostracism, and nurses’ well-being, important gaps remain in understanding how these factors are interconnected within the nursing work context. Existing studies have predominantly examined organizational virtuousness and positive employee outcomes, or workplace ostracism and its adverse associations with nurses’ well-being, as relatively separate lines of inquiry. Consequently, less is known about whether and how workplace ostracism is related to the association between organizational virtuousness and workplace thriving among nurses. The present study addresses this gap by integrating organizational virtuousness, workplace ostracism, and workplace thriving within a single analytical framework and specifically examining the moderating role of workplace ostracism in the organizational virtuousness–workplace thriving relationship. This approach extends current knowledge by considering positive organizational characteristics and negative interpersonal workplace experiences simultaneously rather than examining them in isolation. Theoretically, the study draws on the Job Demands–Resources model to provide a framework for understanding organizational virtuousness as a potentially supportive organizational resource and workplace ostracism as a relevant social or interpersonal workplace demand in relation to workplace thriving. Thus, the study contributes a more integrated perspective on the conditions associated with nurses’ workplace thriving and provides a basis for understanding how the strength of the association between a positive organizational context and thriving may differ across levels of workplace ostracism (Figure 1. Hypothesized model).

Aim of the Study: The current study aimed to determine the relationship between organizational virtuousness and workplace thriving and explore the moderating role of workplace ostracism as perceived by nurses.

## 2. Method

### 2.1. Research Design

A descriptive correlational research design with regression-based moderation analysis was used to examine the relationships between organizational virtuousness, workplace ostracism, and workplace thriving among nurses. This design was appropriate because the study investigated naturally occurring relationships without manipulating study variables. An experimental design was not selected because manipulating workplace experiences was impractical and potentially raised ethical concerns. A longitudinal design was also not adopted because the study aimed to examine these relationships at a single point in time rather than assess changes over time. Furthermore, the cross-sectional approach was more feasible in terms of time and resources while allowing the proposed associations and moderation effect to be examined within the study population. Nevertheless, this design does not establish causality or determine the direction of the observed relationships.

### 2.2. Research Hypotheses

**H1.**  *Organizational virtuousness is positively associated with workplace thriving as perceived by nurses at selected hospitals.*

**H2.**  *Organizational virtuousness is negatively associated with workplace ostracism as perceived by nurses at selected hospitals.*

**H3.**  *Workplace ostracism is negatively associated with workplace thriving as perceived by nurses at selected hospitals.*

**H4.**  *Workplace ostracism moderates the relationship between organizational virtuousness and workplace thriving as perceived by nurses at selected hospitals.*

### 2.3. Setting

The study was conducted at two large hospitals that provide mainly tertiary healthcare services: Shebin El-kom Teaching Hospital and El-Helal Insurance Hospital. These settings were selected because they provided access to the target nursing population and to inpatient and critical care units in which nurses experience the organizational and interpersonal work conditions addressed by the study variables. Including two hospital settings also allowed the study to capture nurses working within more than one hospital context rather than relying on a single institutional setting. The study was therefore conducted in the actual work environment in which organizational virtuousness, workplace ostracism, and workplace thriving are experienced by nurses.

### 2.4. Subjects

A simple random sampling technique was used to select 530 nurses working in the inpatient departments and critical care units of the two participating hospitals who met the eligibility criteria and agreed to participate. A sampling frame of eligible nurses was used, and a computer-generated random list was used to select participants, thereby giving eligible nurses an equal opportunity of selection and reducing the risk of investigator selection bias. The use of simple random sampling was appropriate because the study sought to obtain a sample from the defined population of eligible nurses while minimizing systematic selection based on individual characteristics or availability. The final sample comprised nurses from both participating hospitals and from the specified clinical areas.

The computer-generated selection procedure was applied to the eligible sampling frame rather than to an unrestricted list of nurses. Eligible nurses were identified according to the predefined inclusion criteria, assigned sampling identifiers, and selected according to the computer-generated random sequence until the required number of 530 nurses was reached. This procedure provided a transparent and reproducible method of participant selection and reduced the potential for convenience-based recruitment.

Sample size: G*Power 3.1 was used to conduct a priori power analysis for a multiple regression model including an interaction term (moderation analysis). Assuming a small effect size (f^2^ = 0.02), a significance level of 0.05, and statistical power of 0.80, the minimum required sample size was 395 participants. A total of 530 questionnaires were distributed to allow for potential nonresponse or unusable questionnaires. Of these, 66 questionnaires were discarded because of incomplete responses, resulting in 464 usable questionnaires and an 87.5% response rate. The final sample therefore exceeded the minimum sample size required by the a priori power analysis and provided adequate power for testing the proposed moderation effect.

Inclusion criteria were: (1) staff nurses with more than one year of professional experience; (2) currently working in an inpatient department or critical care unit in one of the participating hospitals; and (3) willingness to participate in the study. The requirement of more than one year of experience was used to ensure that participants had sufficient exposure to their organizational work environment to provide informed perceptions of organizational virtuousness, workplace ostracism, and workplace thriving. Nurses working in the specified clinical areas were included because these were the study’s predefined clinical settings.

### 2.5. Instruments of Data Collection

Three instruments were employed to collect the study data. Instrument selection was based on the conceptual alignment of each measure with one of the study’s variable constructs and on the multidimensional coverage provided by the selected scales. A personal and professional characteristics section collected hospital name, age, gender, marital status, educational background, years of experience, and area of employment. The three study instruments were selected as follows:

Instrument I: The Organizational Virtuousness Scale, developed by [21], was selected to assess organizational virtuousness because it operationalizes the construct through five theoretically relevant dimensions: organizational optimism, trust, compassion, solidarity, and mercy. The scale contains 15 items, with three items representing each dimension. Responses are rated on a five-point Likert scale ranging from 1 (very low) to 5 (very high). Its multidimensional structure provided coverage of the positive organizational qualities examined in the present study.

The total score of organizational virtuousness ranges from 15 to 75, with scores categorized as follows: 15–37 points indicate a low level, 38–56 points indicate a moderate level, and 57–75 points indicate a high level. This scoring system was developed by the researchers using the equal width interval method for systematic categorization.

Instrument II: The Thriving from Work Questionnaire, developed by [55], was selected to assess workplace thriving because it captures thriving as a multidimensional work-related construct rather than as a single aspect of nurses’ well-being. The 30-item questionnaire covers six domains: psychological and emotional well-being (6 items), social well-being (5 items), work–life integration (5 items), basic needs for thriving (4 items), job design and experience (5 items), and safety and physical and mental well-being (5 items). Responses are measured on a five-point Likert scale ranging from 1 (Never) to 5 (Always). This broad domain coverage was considered appropriate for assessing nurses’ overall experience of thriving at work.

The total score for workplace thriving ranges from 30 to 150, categorized as follows: scores of 30–74 indicate a low level, 75–112 a moderate level, and 113–150 a high level of workplace thriving. This scoring system was developed by the researchers using the equal width interval method to ensure systematic and transparent categorization of scores.

Instrument III: The Workplace Ostracism (WPO) Scale, developed by [24], was selected to measure nurses’ perceptions of workplace ostracism. The scale contains 10 items and uses a five-point Likert scale ranging from 1 (strongly disagree) to 5 (strongly agree). The instrument was selected because it directly assesses the experience of being ignored, excluded, or socially disconnected in the workplace, which corresponds to the moderator examined in the present study.

The workplace ostracism total score ranges from 10 to 50. Scores of 10–24 indicate a “low level,” 25–37 represent a “moderate level,” and 38–50 are classified as a “high level” of workplace ostracism. This scoring system, developed by the researchers using the equal width interval method, ensures systematic and transparent categorization of scores.

### 2.6. Validity of the Study Instruments

#### Translation and Back-Translation

The instruments were translated from English into Arabic using a translation and back-translation procedure to support linguistic equivalence, understandability, and cultural appropriateness for the study participants.

1. Initial Translation: A skilled translator who is fluent in Arabic and English and aware of the subtle cultural differences between the two languages translated the instruments into Arabic.
2. Back-Translation: The Arabic version was independently translated back into English by several translators who were fluent in both languages and were not engaged in the original translation. This stage aids in verifying the translation’s accuracy and uniformity.
3. Comparison and Revision: A comparison was made between the back-translated English version and the original English version. Any disagreements were examined and settled by a panel of experts.

Moreover, an exploratory factor analysis (EFA) confirmed the construct validity of the three translated study instruments: the Organizational Virtuousness Scale (15 items), Workplace Thriving Scale (30 items), and Workplace Ostracism Scale (10 items). The Organizational Virtuousness Scale showed a Kaiser–Meyer–Olkin (KMO) measure of 0.724, with Bartlett’s Test statistically significant (χ^2^ = 3472.461, *p* < 0.001) and an explained variance of 63.62%. The Workplace Thriving Scale had a KMO of 0.784, significant Bartlett’s Test (χ^2^ = 8552.217, *p* < 0.001), and explained variance of 64.5%. The Workplace Ostracism Scale exhibited excellent sampling adequacy (KMO = 0.916), significant Bartlett’s Test (χ^2^ = 2377.436, *p* < 0.001), with explained variance at 53.9%. These findings provided evidence that the data were suitable for factor analysis and supported the construct validity of the translated instruments.

### 2.7. Reliability Test for the Study Instruments

Internal consistency reliability was assessed using Cronbach’s alpha. Cronbach’s alpha was calculated for each subscale and for the complete set of items comprising each study instrument; therefore, the total alpha coefficients in Table 1 represent all items within the corresponding instrument. The Organizational Virtuousness Scale demonstrated alpha coefficients ranging from 0.755 to 0.834 across its five dimensions, with a total alpha of 0.864 for all 15 items. The Workplace Thriving Questionnaire demonstrated alpha coefficients ranging from 0.801 to 0.840 across its six domains, with a total alpha of 0.871 for all 30 items. The Workplace Ostracism Scale demonstrated a total alpha of 0.898 for all 10 items. These coefficients indicate a satisfactory to high internal consistency of the study measures.

### 2.8. Pilot Study

A pilot study was conducted with 46 staff nurses (27 from Shebin El-kom Teaching Hospital and 19 from El-Helal Insurance Hospital) to assess the feasibility, clarity, applicability, and estimated completion time of the questionnaires before the main data collection. The pilot participants were not included in the final study sample or statistical analysis. No study items were removed following the pilot study, and the final instruments retained the same item numbers and response formats. The average completion time observed during the pilot informed the estimated 25–40 min required for completion during the main study.

### 2.9. Data Collection Procedure

The hospital management obtained formal written consent, while the staff nurses provided verbal consent. Each nurse required 25–40 min to complete the questionnaires, with data collected during break time over the morning and afternoon shifts. The data collection occurred from 1 March 2024, to 30 April 2024.

### 2.10. Ethical Considerations

The study adhered to ethical research standards by ensuring participant rights. This included voluntary participation, informed consent detailing the study’s purpose, duration, potential benefits, data collection methods, expected outcomes, and the right to withdraw at any time if rights were violated.

The respondent was assured of strict confidentiality in data handling through coding, anonymity was preserved, and the study protocol received approval from the ethical and research committee at the Faculty of Nursing, Menoufia University, prior to commencement.

### 2.11. Statistical Design

Data were analyzed using SPSS version 26.0. Descriptive statistics were used to summarize the sample and study variables, including means, standard deviations (SDs), frequencies, and percentages as appropriate. Before inferential analysis, the distributional assumptions for parametric analyses were assessed using the Kolmogorov–Smirnov and Shapiro–Wilk tests. Pearson’s correlation coefficient was used to examine the direction and strength of the bivariate relationships among organizational virtuousness, workplace ostracism, and workplace thriving because the study variables were treated as continuous measures.

Before conducting the regression-based moderation analysis, multicollinearity among the predictor variables and covariates included in the regression model was assessed using tolerance and variance inflation factor (VIF) statistics. The results showed that organizational virtuousness had a tolerance value of 0.5260 and a VIF of 1.9010, while workplace ostracism had a tolerance value of 0.3087 and a VIF of 3.2390. Among the control variables, age had a tolerance value of 0.4899 and a VIF of 2.0412, gender had a tolerance value of 0.9528 and a VIF of 1.0495, qualification had a tolerance value of 0.6116 and a VIF of 1.6450, and years of experience had a tolerance value of 0.4618 and a VIF of 2.1653. The VIF values ranged from 1.0495 to 3.2390, and all were below 5, indicating no evidence of severe multicollinearity among the variables assessed.

A regression-based moderation analysis was then performed using PROCESS macro Model 1 to test whether workplace ostracism moderated the relationship between organizational virtuousness (predictor) and workplace thriving (outcome). Age, gender, qualification, and years of experience were entered as covariates in the moderation model. The interaction term between organizational virtuousness and workplace ostracism was evaluated to determine whether the strength of the predictor–outcome relationship varied according to the level of workplace ostracism. Statistical significance was evaluated using two-sided tests with *p* < 0.01 and 95% confidence intervals (CIs). The moderation analysis was interpreted using the regression coefficient of the interaction term, its confidence interval, the change in explained variance (ΔR^2^), and the conditional effect of organizational virtuousness on workplace thriving at different levels of workplace ostracism.

## 3. Results

Table 2 indicates that 47.8% of the study sample was aged 35 to 45, predominantly female (84.1%) and married (64%). One-third (33.8%) held a nursing diploma, and over half had 10 to less than 15 years of experience, with 55% employed in the inpatient department of Shebin El-kom Teaching Hospital and El-Helal Insurance Hospital.

**Figure 2:**
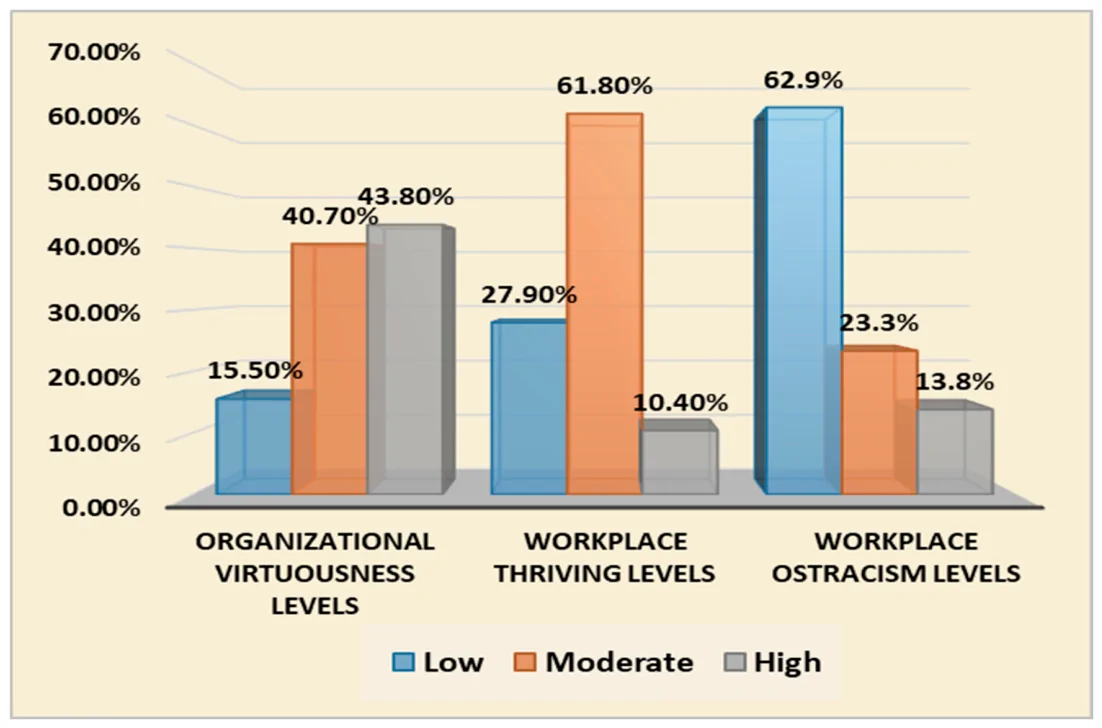
Distribution of levels of organizational virtuousness, workplace thriving, and workplace ostracism as perceived by nurses (*n* = 464).

Figure 2 shows that 43.80% of staff nurses view their organization as having high virtuousness. In contrast, 61.80% experience moderate workplace thriving, while nearly 62.9% report low levels of workplace ostracism.

Table 3 indicates that the mean score for organizational virtuousness reported by staff nurses was 51.12 ± 12.76, with organizational optimism ranking highest at 71.53% and organizational trust lowest at 62.6%. The total mean score for workplace thriving was 86.65 ± 24.69, led by psychological and emotional well-being at 68.59%, while job design and work–life integration ranked lowest at 52.88%. The mean score for workplace ostracism was 25.50 ± 8.90, out of a maximum of 50.00.

Table 4 represents that there was a statistically significant positive correlation between organizational virtuousness and workplace thriving (r = 0.817, *p* = 0.000). Moreover, there was a statistically significant negative correlation between organizational virtuousness and workplace ostracism (r = −0.650, *p* = 0.000). Also, there was a statistically significant negative correlation between workplace thriving and workplace ostracism (r = −0.549, *p* = 0.000).

Table 5 presents the results of the regression-based moderation analysis examining whether workplace ostracism was associated with variation in the relationship between organizational virtuousness and workplace thriving. After controlling for age, gender, qualification, and years of experience, the overall regression model was statistically significant, F (7 456) = 182.7278 and *p* < 0.001, and accounted for 73.72% of the variance in workplace thriving (R^2^ = 0.7372). Organizational virtuousness was significantly and positively associated with workplace thriving (B = 0.3317, *p* < 0.001), whereas workplace ostracism was significantly and negatively associated with workplace thriving (B = −0.2351, *p* < 0.001). The interaction between organizational virtuousness and workplace ostracism was also statistically significant and positive (B = 0.0070, SE = 0.0016, t = 4.4124, *p* < 0.001, 95% CI [0.0039, 0.0102]), indicating that the strength of the observed association between organizational virtuousness and workplace thriving varied according to the level of workplace ostracism.

Moreover, the interaction term accounted for a significant incremental proportion of variance in workplace thriving (ΔR^2^ = 0.0108, F = 17.7385, *p* < 0.001), demonstrating that the interaction contributed explanatory value beyond the main effects and control variables.

**Figure 3:**
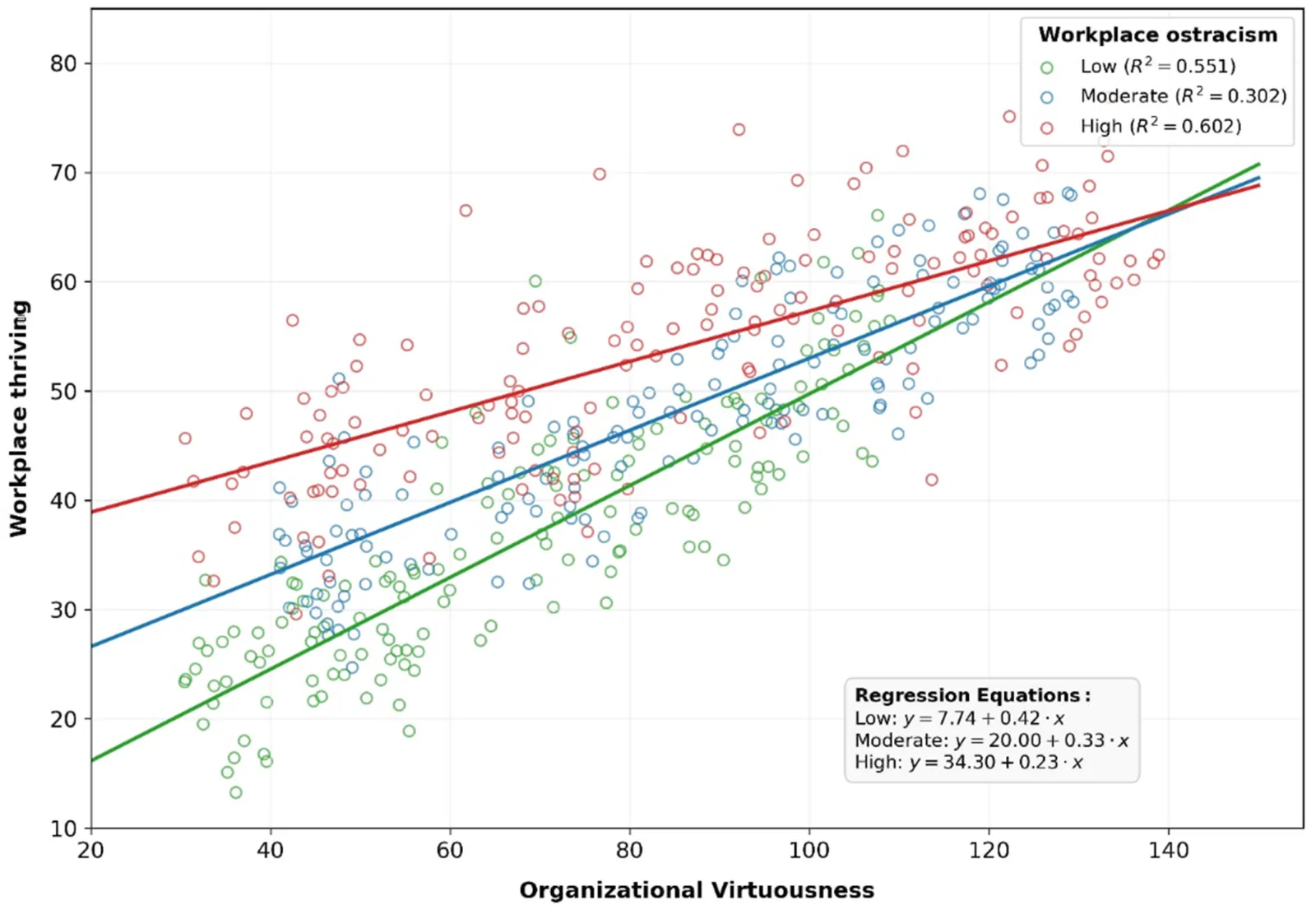
Moderation analysis of the relationship between organizational virtuousness and workplace thriving by workplace ostracism (*n* = 464).

As illustrated in Figure 3, the positive association between organizational virtuousness and workplace thriving becomes progressively stronger as the level of workplace ostracism increases, as indicated by the steeper slope under high workplace ostracism compared with moderate and low levels. Thus, although workplace ostracism is independently associated with lower workplace thriving, its positive interaction with organizational virtuousness suggests that organizational virtuousness may become particularly important for supporting nurses’ workplace thriving under conditions of greater social exclusion. This finding highlights the potential role of a virtuous organizational environment characterized by fairness, trust, respect, compassion, and supportive relationships as an important organizational resource in socially adverse workplace conditions.

Table 6 revealed a positive and statistically significant association between organizational virtuousness and workplace thriving at all three levels of workplace ostracism (*p* < 0.001). Specifically, the regression coefficient increased from 0.2695 at low levels of ostracism to 0.3317 at moderate levels and 0.3939 at high levels. All confidence intervals excluded zero, confirming the statistical significance of these conditional effects. Consistent with the significant interaction effect reported in the moderation analysis (B = 0.0070, *p* < 0.001), these findings indicate that the positive association between organizational virtuousness and workplace thriving became stronger as workplace ostracism increased, supporting its moderating role.

## 4. Discussion

The present study examined the relationships among organizational virtuousness, workplace thriving, and workplace ostracism among nurses, with particular attention to the moderating role of workplace ostracism in the relationship between organizational virtuousness and workplace thriving. Overall, the findings supported the proposed relationships and highlight the importance of positive organizational conditions and interpersonal experiences in relation to nurses’ workplace functioning. Importantly, the findings indicate that organizational virtuousness and workplace ostracism are closely related to workplace thriving and should be considered together when examining nurses’ workplace experiences, as the interpersonal climate within an organization may be relevant to the strength of the association between positive organizational characteristics and nurses’ reported experiences of thriving.

Before discussing the hypotheses, it is noteworthy that most participants were female, married, and had between 10 and 15 years of nursing experience. These characteristics are broadly consistent with the demographic profile commonly observed in nursing populations. However, demographic variables were not significantly associated with workplace thriving in the moderation model. This finding suggests that thriving may be more closely related to nurses’ perceptions of their work environment and psychosocial resources than to demographic characteristics. Thus, regardless of individual background, nurses may be more likely to report thriving when they perceive their work environments as characterized by supportive relationships, positive organizational practices, and opportunities for learning and personal development. This interpretation is consistent with the contemporary thriving literature emphasizing the role of workplace conditions and organizational resources in relation to vitality and learning at work [30,56].

**H1.**  *Organizational virtuousness is positively associated with workplace thriving as perceived by nurses at selected hospitals.*

From the perspective of the Job Demands–Resources model, this finding may be understood in relation to the supportive organizational resources available to nurses. Organizational virtuousness may represent a positive organizational context that is relevant to nurses’ workplace thriving, particularly in a work environment characterized by substantial interpersonal and emotional demands. Furthermore, the findings supported the first hypothesis, as organizational virtuousness was significantly and positively associated with workplace thriving (r = 0.817, *p* < 0.001) and remained significantly associated with thriving in the moderation model (β = 0.332, *p* < 0.001). This finding indicates that nurses who perceived greater organizational virtuousness also tended to report higher levels of workplace thriving. The strength of this association suggests that positive organizational characteristics may be closely related to the social and psychological context in which nurses experience vitality and learning.

From the perspective of positive organizational scholarship, organizational virtuousness encompasses organizational qualities such as optimism, trust, compassion, solidarity, and forgiveness that are associated with positive social interactions and psychological resources [21]. These characteristics may be particularly relevant to nursing because nurses routinely encounter high workloads, emotionally demanding patient situations, complex teamwork requirements, and situations requiring sustained interpersonal engagement. In such circumstances, perceptions that the organization is compassionate, trustworthy, and supportive may be related to nurses’ reported psychological energy and openness to learning and professional development. Thus, the relationship between organizational virtuousness and thriving may reflect the coexistence of positive organizational characteristics and relational and psychological resources that are relevant to vitality and learning.

The present findings are consistent with [57] who reported that favorable organizational culture was associated with nurses’ thriving through emotional commitment and work engagement. Their findings suggest that positive organizational environments are related to employees’ attachment to their organizations and active engagement in work, which may also be associated with thriving. Similarly, Shen, et al., (2024) [19] demonstrated the importance of thriving in relation to favorable professional outcomes among clinical nurses, while [56] reported that flourishing mediated the relationship between structural empowerment and important workforce outcomes. Collectively, these findings indicate that positive organizational environments are associated with nurses’ ability to perform their roles while also experiencing psychological growth and development.

Notably, the correlation between organizational virtuousness and workplace thriving in the present study was exceptionally high (r = 0.817). This finding warrants particular consideration rather than being interpreted solely as evidence of a strong positive relationship. One possible explanation is the substantial conceptual proximity between the two constructs. Organizational virtuousness reflects positive organizational characteristics and relationships, whereas thriving reflects employees’ experiences of vitality and learning. An organizational environment characterized by trust, compassion, solidarity, and optimism may therefore be closely related to the interpersonal and psychological conditions under which employees report feeling energetic, supported, and capable of learning. In this sense, the constructs are theoretically distinct but may be closely connected in nurses’ lived workplace experiences.

A second possible explanation is the nature of nursing work. Nurses depend heavily on teamwork, interpersonal support, communication, and collaboration to accomplish their responsibilities. Consequently, organizational virtues may be experienced by nurses not as abstract organizational characteristics but through everyday interactions with colleagues, supervisors, and the wider organization. When such interactions are consistently positive, nurses may simultaneously perceive their organization as virtuous and report greater vitality and learning, resulting in a particularly strong observed association.

Nevertheless, methodological factors should also be considered when interpreting the magnitude of this correlation. Both constructs were assessed using self-administered measures based on nurses’ perceptions and collected within the same study context. Shared method variance and respondents’ general tendency to evaluate different aspects of their work environment consistently in a positive or negative direction may therefore have contributed to the strength of the association. In addition, the cross-sectional correlational nature of the analysis does not permit causal interpretation. It cannot be concluded that organizational virtuousness directly causes workplace thriving; rather, the findings demonstrate a strong empirical association between nurses’ perceptions of these two constructs. Future longitudinal and multi-source studies are therefore needed to examine temporal associations and to further establish the distinctiveness of the two constructs.

**H2.**  *Organizational virtuousness is negatively associated with workplace ostracism as perceived by nurses at selected hospitals.*

The second hypothesis was supported, as organizational virtuousness was significantly and negatively associated with workplace ostracism (r = −0.650, *p* < 0.001). Nurses who perceived greater organizational virtuousness tended to report lower levels of social exclusion and neglect. This relationship may be understood in light of the interpersonal characteristics associated with organizational virtuousness. Organizational solidarity is conceptually related to inclusion and cooperation, trust is relevant to positive interpersonal relationships, and compassion reflects sensitivity to coworkers’ needs. Collectively, these characteristics may be associated with fewer perceptions of exclusionary interpersonal experiences.

From the perspective of positive organizational scholarship, organizational virtuousness emphasizes positive organizational qualities and practices that may be relevant to employees’ workplace experiences and interpersonal relationships [58]. In nursing settings, attributes such as trust, compassion, and solidarity may be associated with lower levels of perceived social exclusion. Nevertheless, this interpretation remains tentative, as the present findings do not establish that organizational virtuousness prevents workplace ostracism.

This finding is consistent with the principles of positive organizational scholarship, which emphasize the relevance of positive organizational characteristics to social relationships and constructive interpersonal behaviors [21]. In nursing environments, where effective teamwork is closely connected to the coordination and safety of patient care, an organizational culture characterized by compassion, trust, and solidarity may be associated with stronger interpersonal relationships and fewer experiences of exclusion.

However, the observed negative association should not be interpreted as evidence that organizational virtuousness alone prevents workplace ostracism. Ostracism may be associated with multiple interpersonal and organizational circumstances, including interpersonal conflicts, communication problems, workload pressures, group dynamics, perceived competition, or differences in status and power. Therefore, even organizations perceived as relatively virtuous may experience episodes of exclusion. The finding that workplace ostracism was present despite generally favorable perceptions of organizational virtuousness supports the importance of addressing ostracism explicitly rather than assuming that a positive organizational culture is sufficient to eliminate it.

**H3.**  *Workplace ostracism is negatively associated with workplace thriving as perceived by nurses at selected hospitals.*

The third hypothesis was also supported. Workplace ostracism was significantly and negatively associated with workplace thriving (r = −0.549, *p* < 0.001), and it remained significantly and negatively associated with thriving in the moderation model (β = −0.235, *p* < 0.001). This finding indicates that nurses who reported greater exclusion also tended to report lower levels of thriving, characterized by vitality and learning.

The negative association can be interpreted in light of the need-threat model of ostracism, which proposes that exclusion is associated with threats to fundamental psychological needs, including belongingness, self-esteem, control, and meaningful existence [59]. When nurses perceive that colleagues or others in the workplace ignore, exclude, or socially isolate them, such experiences may be accompanied by psychological strain and reduced availability of cognitive and emotional resources for learning, engagement, and professional development. Ostracism may therefore be related to lower thriving through reduced participation, communication, help-seeking, and engagement with workplace learning opportunities. However, these potential mechanisms remain theoretical interpretations rather than causal pathways established by the present study.

Recent nursing research provides additional support for this interpretation. Elliethey, et al., (2024) [60] reported an association between workplace ostracism and counterproductive work behaviors among nurses. Attia, et al., (2025) [61] similarly demonstrated relationships between workplace exclusion, protective silence, and emotional exhaustion, while Ali, et al., (2025) [62] found that workplace ostracism was associated with poorer nursing care quality and reduced creative work behavior. These findings suggest that ostracism is related not only to interpersonal experiences but also to employees’ psychological functioning, work behaviors, and organizational contributions.

An alternative explanation should nevertheless be considered. The relationship between ostracism and reduced thriving may potentially be reciprocal. Nurses who experience lower vitality, reduced engagement, or fewer opportunities for learning may participate less in social and professional interactions and could consequently perceive greater social exclusion. Therefore, although the present findings are consistent with an association between ostracism and lower thriving, the correlational design does not establish the direction of this relationship. Longitudinal research would help clarify whether workplace ostracism is temporally associated with subsequent changes in thriving, whether low thriving is associated with subsequent perceptions of ostracism, or whether both processes may co-occur over time.

**H4.**  *Workplace ostracism moderates the relationship between organizational virtuousness and workplace thriving as perceived by nurses at selected hospitals.*

The fourth hypothesis was supported, as workplace ostracism significantly moderated the relationship between organizational virtuousness and workplace thriving. The interaction between organizational virtuousness and workplace ostracism was statistically significant (B = 0.0070, SE = 0.0016, t = 4.4124, *p* < 0.001, 95% CI [0.0039, 0.0102]). Furthermore, the addition of the interaction term significantly improved the explanatory power of the model (ΔR^2^ = 0.0108, F-change = 17.7385, *p* < 0.001). The full model explained 73.72% of the variance in workplace thriving (R^2^ = 0.7372), indicating substantial explanatory power.

Importantly, the moderation finding requires a nuanced interpretation. Workplace ostracism had a significant negative main role in the statistical model (B = −0.2351, *p* < 0.001), indicating that higher levels of ostracism were statistically associated with lower levels of workplace thriving. However, because the study design does not establish temporal or causal relationships, this coefficient should not be interpreted as evidence that ostracism causes reductions in thriving. The positive interaction coefficient (B = 0.0070) indicates that the strength of the positive association between organizational virtuousness and workplace thriving varied according to the level of workplace ostracism. Thus, workplace ostracism did not simply eliminate the observed positive association between organizational virtuousness and thriving. Rather, the statistical association between the two constructs differed significantly across levels of ostracism. This finding indicates that workplace ostracism may shape the extent to which a virtuous organizational environment translates into employees’ positive functioning and thriving at work.

One possible interpretation is that organizational virtuousness may be particularly salient to nurses who report greater interpersonal difficulties at work. In contexts where nurses perceive exclusion or social neglect, organizational expressions of compassion, solidarity, trust, and optimism may be perceived as relatively more meaningful because they represent broader organizational characteristics beyond immediate interpersonal experiences. Consequently, nurses experiencing greater ostracism may report a stronger association between perceived organizational virtuousness and thriving, even though their overall level of thriving may remain lower in the presence of higher reported ostracism. In this sense, the observed pattern may be consistent with organizational virtuousness functioning as a potentially relevant organizational resource, although the present design cannot establish a compensatory causal process.

The interaction finding may be understood through the lens of the Job Demands–Resources (JD-R) model [63], which explains how workplace resources and demands relate to employees’ work experiences. In the present study, organizational virtuousness can be conceptualized as a favorable organizational resource, while workplace ostracism represents a potentially adverse interpersonal demand. The significant interaction suggests that the association between organizational virtuousness and workplace thriving differs depending on the level of workplace ostracism experienced. By examining these organizational and interpersonal factors together, the findings offer a context-specific application of the JD-R model to nursing. However, this theoretical interpretation should not be taken as evidence of a causal relationship or as confirmation that one factor offsets the other.

This finding also provides an important refinement of the interpretation of organizational virtuousness. Positive organizational values may have different meanings depending on nurses’ interpersonal experiences. Nurses who report little ostracism may already perceive considerable relational support within their immediate work environment, whereas those reporting greater exclusion may place greater importance on broader organizational resources and values. Thus, organizational virtuousness may be particularly salient when interpersonal relationships within the workplace are perceived as less supportive.

Nevertheless, caution is required when interpreting this interaction. The interaction term accounted for an additional 1.08% of the variance in workplace thriving, indicating that although the moderating effect was statistically significant, its incremental contribution was relatively modest. In addition, the present correlational design does not establish causal or temporal relationships among the variables. The findings therefore should not be interpreted as evidence that organizational virtuousness causes nurses to thrive more under conditions of ostracism. Rather, they indicate that the association between perceived organizational virtuousness and workplace thriving differs according to nurses’ perceived level of workplace ostracism.

Overall, the findings suggest that healthcare organizations may benefit from a dual strategy that addresses both organizational virtuousness and workplace ostracism. Promoting compassion, trust, solidarity, and psychological safety may be relevant to nurses’ perceptions of their organizational environment; however, these efforts should be accompanied by proactive measures to identify exclusionary behaviors, strengthen inclusive teamwork, and ensure that all nurses have equitable opportunities for participation and support. Given the correlational nature of the present study, these recommendations should be considered as organizational strategies informed by the observed associations rather than interventions whose causal effectiveness has been established.

### Limitations

Several limitations should be considered when interpreting the findings. First, the study relied on self-administered, self-report questionnaires, which may have introduced response bias and common method variance because the study variables were assessed using participants’ perceptions within the same data-collection context. To minimize these potential sources of bias, participants were provided with standardized instructions, informed that their responses would be treated confidentially, and encouraged to provide honest responses without identifying information. Nevertheless, the possibility of response and common method bias cannot be completely excluded. Future studies should consider using multiple data sources, objective indicators, longitudinal designs, or data collected at different time points to reduce reliance on a single self-report source.

Second, the study was conducted among nurses within a specific geographical and healthcare context, which may limit the generalizability of the findings to nurses working in other regions, healthcare organizations, or clinical settings. Future research involving multiple healthcare institutions and different geographical areas would enhance the diversity of the study context and allow comparison across organizational and healthcare environments.

Third, the cross-sectional design limits the ability to establish temporal or causal relationships among organizational virtuousness, workplace ostracism, and workplace thriving. Although significant associations and a moderation effect were identified, the directionality of these relationships cannot be determined from the present data. Longitudinal studies are therefore recommended to examine how these variables are related over time and to provide stronger evidence regarding their temporal ordering.

Fourth, although the moderation effect was statistically significant, the interaction term accounted for a relatively small additional proportion of the variance in workplace thriving (ΔR^2^ = 0.0108). Thus, the statistical significance of the interaction should be considered alongside its modest incremental contribution. Future research could examine additional contextual and interpersonal factors that may further explain variations in the relationship between organizational virtuousness and workplace thriving.

Finally, the relatively limited empirical literature specifically examining the moderating role of workplace ostracism in the relationship between organizational virtuousness and workplace thriving restricted direct comparison with previous findings. The limited number of comparable studies also highlights the need for further research using longitudinal, multi-site, and multi-source designs to replicate the present findings and further clarify the nature of these associations.

## 5. Practical Implications

The findings provide several practical directions for nurse managers and healthcare organizations. First, nursing managers may incorporate brief assessments of workplace ostracism into routine staff feedback or employee-engagement activities to identify nurses who may be experiencing social exclusion. Clear and confidential mechanisms for reporting exclusionary, disrespectful, or discriminatory behaviors may also be established, with defined procedures for follow-up and appropriate managerial response.

Second, nurse managers may use team-based strategies to strengthen inclusive workplace interactions. These may include structured team-building activities, communication skills training, conflict-resolution sessions, and regular opportunities for staff to raise interpersonal concerns. Particular attention may be given to nurses who report feeling excluded, including providing access to supervisory support, mentoring, or facilitated communication when appropriate.

Third, nurse-manager development programs may incorporate competencies related to inclusive leadership, respectful communication, compassion, trust-building, and management of interpersonal conflict. At the organizational level, values such as compassion, trust, solidarity, and optimism may be incorporated into leadership development, staff orientation, and workplace policies to provide practical guidance for interpersonal conduct and teamwork.

Finally, organizations may monitor organizational virtuousness, workplace ostracism, and workplace thriving through periodic staff surveys or quality-improvement activities. Such monitoring may help managers identify areas requiring attention and evaluate whether organizational initiatives are accompanied by changes in nurses’ reported workplace experiences. These recommendations are based on the associations observed in the present study and should not be interpreted as evidence of causal effectiveness. Future longitudinal and intervention studies are needed to determine whether specific organizational strategies are associated with sustained improvements in workplace thriving and other workforce outcomes.

## 6. Conclusions

The present study demonstrated significant associations among organizational virtuousness, workplace ostracism, and workplace thriving among nurses. Organizational virtuousness was strongly and positively associated with workplace thriving (r = 0.817, *p* < 0.0001), whereas it was significantly and negatively associated with workplace ostracism (r = −0.650, *p* < 0.0001). Workplace ostracism was also significantly and negatively associated with workplace thriving (r = −0.549, *p* < 0.0001).

In the moderation model, organizational virtuousness remained positively associated with workplace thriving (B = 0.3317, *p* < 0.0001), while workplace ostracism remained negatively associated with workplace thriving (B = −0.2351, *p* = 0.0001). The organizational virtuousness × workplace ostracism interaction was statistically significant (B = 0.0070, SE = 0.0016, t = 4.4124, *p* < 0.0001, 95% CI [0.0039, 0.0102]), indicating that the strength of the association between organizational virtuousness and workplace thriving varied according to workplace ostracism. The interaction accounted for an additional 1.08% of the variance in workplace thriving (ΔR^2^ = 0.0108, F-change = 17.7385, *p* < 0.0001), while the overall model explained 73.72% of the variance in workplace thriving (R^2^ = 0.7372, F(7, 456) = 182.7278, *p* < 0.0001).

Overall, the findings indicate that organizational virtuousness and workplace ostracism are significantly associated with workplace thriving and that their relationships are statistically interconnected within the observed sample. Given the cross-sectional correlational design, these findings represent statistical associations and should not be interpreted as evidence of temporal or causal relationships. Future longitudinal and intervention-based studies are warranted to examine the temporal pattern of these relationships and to determine whether targeted organizational strategies are associated with changes in workplace thriving over time.

## Figures and Tables

**Table 1:** Cronbach alpha coefficients for study variables.

Study Variable	No. of Items	Cronbach’s Alpha (α)
Organizational Optimism	3 items	0.755
Organizational Trust	3 items	0.796
Organizational Compassion	3 items	0.784
Organizational Solidarity	3 items	0.834
Organizational Mercy	3 items	0.822
Total Organizational Virtuousness	15 items	0.864
Psychological and Emotional Well-Being	6 items	0.840
Social Well-Being from Work	5 items	0.839
Work–Life Integration	5 items	0.803
Basic Needs from Thriving from Work	4 items	0.813
Job Design and Experience from Work	5 items	0.822
Safety and Physical Well-Being from Work	5 items	0.801
Total Workplace Thriving	30 items	0.871
Total Workplace Ostracism Scale	10 items	0.898

Note. Cronbach’s alpha was calculated for each subscale and for the complete set of items of each instrument. The total coefficients represent all items in the corresponding instrument.

**Table 2:** Personal characteristics of the subjects studied (*n* = 464).

	Variables	No.	%
Age			
	15–<25 years	7	1.5
	25–<35 y	149	32.2
	35–<45 y	222	47.8
	45 or more	86	18.5
Gender			
	Male	74	15.9
	Female	390	84.1
Marital status			
	Single	97	20.9
	Married	297	64
	Divorced	52	11.2
	Widow	18	3.9
Hospital			
	Shebin El-kom Teaching	270	58.2
	El-Helal Insurance	194	41.8
Qualification			
	Diploma in nursing	157	33.8
	Associate degree in nursing	153	33.0
	Bachelor’s degree in nursing	154	33.2
Experience years			
	<5 years	50	10.7
	5–<10 y	60	13
	10–< 15 y	256	55.2
	15 or more	98	21.1
Area of work			
	Critical care unit	209	45.0
	Inpatient department	255	55.0
	Total	464	100

**Table 3:** Descriptive statistics of organizational virtuousness, workplace thriving, workplace ostracism and their dimensions among nurses (*n* = 464).

Study Variables	Mean	SD	Max	Min	Mean%	Ranking
Total Organizational Virtuousness	51.12	12.76	75.00	15.00	68.16	
- Organizational Optimism	10.73	2.74	15.00	3.00	71.53	1
- Organizational Trust	9.39	2.84	15.00	3.00	62.6	5
- Organizational Compassion	10.04	2.83	15.00	3.00	66.933	4
- Organizational Solidarity	10.62	3.02	15.00	3.00	70.8	2
- Organizational Mercy	10.34	2.82	15.00	3.00	68.93	3
Total Workplace Thriving	86.65	24.69	146.00	30.00	59.35	
- Psychological and Emotional Well-Being	19.89	5.68	29.00	6.00	68.59	1
- Social Well-Being from Work	15.07	5.01	25.00	5.00	60.28	3
- Work–Life Integration	14.53	4.73	24.00	5.00	60.54	2
- Basic Needs from Thriving from Work	10.65	3.84	20.00	4.00	53.25	4
- Job Design and Experience from Work	13.22	4.86	25.00	5.00	52.88	5
- Safety and Physical Well-Being from Work	13.22	4.83	25.00	5.00	52.88	5
Total Workplace Ostracism (10 Items)	25.50	8.90	50.00	17.00	51	

**Table 4:** Pearson correlation coefficients among organizational virtuousness, workplace ostracism and workplace thriving (*n* = 464).

	Workplace Thriving		Workplace Ostracism	
	r.	*p*. Value	r.	*p*. Value
Organizational Virtuousness	0.817 **	<0.0001	−0.650 **	<0.0001
Workplace Thriving	1	-----	−0.549 **	<0.0001

** Highly statistically significant at *p* < 0.0001.

**Table 5:** Moderation analysis of the relationship between organizational virtuousness and workplace thriving by workplace ostracism (*n* = 464).

Predictor	Coeff	SE	t	*p*	LLCI	ULCI
Constant	50.9732	3.1076	16.4025	0.0000	44.8661	57.0803
Organizational virtuousness	0.3317	0.0152	21.8272	0.0000	0.3018	0.3615
Workplace ostracism	−0.2351	0.0583	−4.0304	0.0001	−0.3497	−0.1205
Int_1	0.0070	0.0016	4.4124	0.0000	0.0039	0.0102
Age	−0.0938	0.5955	−0.1576	0.8748	−1.2641	1.0764
Gender	−0.1071	0.8563	−0.1251	0.9005	−1.7899	1.5756
Qualification	0.2641	0.4775	0.5532	0.5804	−0.6742	1.2025
Experience years	0.3121	0.6735	0.4634	0.6433	−1.0116	1.6357
R	0.8586					
R-sq	0.7372					
F	182.7278					
df1	7.0000					
df2	456.0000					
P	<0.0001					
Int_1						
R2-chng	0.0108					
F	17.7385					
P	<0.0001					

Int_1: interaction of organizational virtuousness and workplace ostracism.

**Table 6:** Conditional effects of organizational virtuousness on workplace thriving at different levels of workplace ostracism (*n* = 464).

Workplace Ostracism	Slope (B)	SE	t	*p*	LLCI	ULCI
Low (−1 SD)	0.2695	0.0200	13.48	<0.001	0.2303	0.3087
Moderate (Mean)	0.3317	0.0152	21.83	<0.001	0.3019	0.3615
High (+1 SD)	0.3939	0.0200	19.70	<0.001	0.3547	0.4331

## Data Availability

The data presented in this study are available from the corresponding authors upon reasonable request due to privacy and ethical restrictions.
